# Addressing the hypothyroidism impasse - seeking a consensus for the treatment of dissatisfied hypothyroid patients

**DOI:** 10.3389/fendo.2025.1640663

**Published:** 2025-09-23

**Authors:** Tim Welborn

**Affiliations:** ^1^ Department of Medicine, University of Western Australia, Nedlands WA, Australia; ^2^ Department of Endocrinology, Sir Charles Gairdner Hospital, Nedlands WA, Australia

**Keywords:** hypothyroidism, persisting symptoms, combination therapy, thyroid hyporeflexia, autoimmunity, psychoneurosis

## Abstract

Physicians demonstrate differing attitudes about the management of hypothyroid patients on optimal replacement with thyroxine (T4) who have persisting symptoms. The use of liothyronine (T3) as combination therapy (T4+T3) has been promoted, and some patients demonstrate a compelling benefit. They can be identified by delayed relaxation of tendon reflexes, that is, a positive Woltman’s sign. This is specific for T3 responders. A generation of Endocrinologists has neglected this useful clinical sign. Patients with autoimmune symptoms and elevated anti-thyroid peroxidase (anti-TPO) antibodies show improvement after total thyroidectomy, but with the risk of significant postoperative complications. This discrete subgroup tends to be disregarded by authorities in the literature. There is a compelling need for further research on the nonsurgical treatment of this subgroup. Many dissatisfied patients have psychosomatic symptoms because of the burden of having a chronic disease, the need to take regular medication, and other psychosocial concerns. They have medically not yet explained symptoms (MNYES) and require support, counselling, and sometimes cognitive behaviour therapy. True thyroxine allergy and/or sensitivity does occur but is extremely rare. These four subgroups could be designated as – (1) T3-hypothyroidism; (2) Anti-TPO-toxic hypothyroidism; (3) Hypothyroidism-in-denial; and (4) Thyroxine- sensitivity. Recognition of these individual categories is necessary to achieve agreement among physicians and to promote further targeted research.

## Introduction

Thyroxine (T4) replacement therapy for overt hypothyroidism is the standard of care. The majority of patients do very well on this medication with resolution of symptoms. Most achieve target laboratory levels of Thyrotropin (TSH) and T4. It is common clinical experience that some patients on optimal thyroxine replacement have persistent distressing symptoms, and this affects an estimated 5% to 20% of all hypothyroid patients (1). Their management is controversial, and there has much prevarication in the medical literature.

## Argument

Two recent papers typify the divergent attitudes of physicians who treat these patients (2, 3). The problem has challenged physicians for decades and it has featured annually in meetings of the European and American Thyroid Associations. There is a pressing need for a consensus that recognises the existence of the discrete subgroups within this population.

The first paper comments on the use and misuse of liothyronine (T3), and concludes that most of these patients belong to the category of having medically not yet explained symptoms (MNYES). This diagnosis is reached by excluding other known causes of symptoms. It is closely related to somatic symptom disorder, and can result in significant disruption of daily life.

The authors advise caution about combination therapy. They state that the number of patients with low tissue T3 is much less common than has been proposed. Improved symptoms *may* be explained by an enhanced sense of well-being associated with T3 therapy.

Hegedus and colleagues also state that the ‘*low T3 hypothesis’* has been tested in 19 randomised controlled trials, and these provided ‘robust’ evidence of the non-superiority of adding T3 as combination therapy. This is true for the *general* hypothyroid population but not those with distressing symptoms. Most would show no change with the addition of T3. A consensus of international thyroid associations has now recommended that future clinical trials select only those hypothyroid patients who are dissatisfied with T4 monotherapy (4).

Many physicians express the view that persistent symptoms are predominantly psychosomatic. The THESIS study (Treatment of Hypothyroidism in Europe by Specialists, an International Survey) reflects the clinical experience of 6,058 European specialists responding to an online questionnaire survey (5). They consider that persistent symptoms are mostly explained by psychosocial factors, unrealistic patient expectations, the burden of chronic disease requiring regular medication, and the chronic fatigue syndrome.

The second paper (3) promotes combination therapy and acknowledges that many patients express a preference for it. In 11 randomised controlled trials involving 1135 patients almost 50% of the patients preferred combination therapy, 25% preferred T4 monotherapy, and 25% had no preference (6). Bianco addresses the frequently stated concern about the safety of T3 supplementation, citing two large meta-analyses of published randomised controlled clinical trials which showed no significant differences in adverse event rates between T4 monotherapy and combination therapy. He also refers to three long-term retrospective studies that excluded any increase in cardiovascular, skeletal, or neurological events, or all-cause mortality, in patients on therapy containing T3 (3). In the setting of shared decision-making, patients should be informed that there are effective and safe options to T4 monotherapy, namely the addition of (low dose) T3 as combination therapy or the use of desiccated thyroid extract (DTE)). In conclusion the author reiterates professional guidelines from the European and American Thyroid Associations that recommend a trial of T4+T3 in patients with clinically relevant residual symptoms (4).

Clearly there is diversity within the subpopulation of patients with persistent symptoms and clearly there is division between the endocrinologists who treat them. This impasse must be addressed, and there is a way forward. Four discrete subgroups can be identified. I have proposed brief descriptive eponyms (7) for each subgroup, as follows.

## T3-hypothyroidism

The low tissue T3 hypothesis has surely been confirmed many times in individual practices with physicians pursuing an ‘N=1’ personal trial. There are only four case reports of patients showing the typical dramatic improvement with combination therapy (7). These cases may show suggestive trends in the pattern of their laboratory tests – although within the normal range – T4 levels are somewhat higher and T3 levels somewhat lower than the mean and there is a high T4 to T3 ratio (8). Many have gone from doctor to doctor and many have had frequent laboratory tests seeking a solution to their problem.

What is important and not widely known is that these patients can be identified prospectively by a simple and highly specific clinical sign. Examination of the tendon reflexes will show the typical delayed relaxation phase of about one second characteristic of florid hypothyroidism, in the presence of normal T4 and TSH levels (8). All upper and lower limb tendon reflexes will show this sign. The preferred method is to test the ankle jerks to elicit a positive Woltman’s sign (9). In this context the plantar tap technique is most convenient (10). With combination therapy the symptoms resolve dramatically and the reflexes become normal. This clinical sign is distinctive, and the use of measuring devices such as the Burdick photomotogram is not necessary. The fact that responders to combination therapy show delayed reflexes was first reported in 2013 (11). Clearly a definitive clinical trial is long overdue. Since the finding is very specific for T3 responders, such a trial could be conducted in a relatively small number of patients over a comparatively short time frame (12). My personal experience of at least 30 cases is that more than half of the ‘unhappy hypothyroid’ population fall into this category. This could reflect a referral bias since I had locally broadcast my views.

With our knowledge of current thyroid hormone physiology, delayed reflexes should be expected when intra-cellular T3 deficiency exists in the presence of normal circulating T4 and TSH levels. T4 is essentially a pro-hormone, and most if not all of the peripheral actions of thyroid hormones are mediated by peripheral intra-cellular conversion of T4 to T3 by deiodinase enzymes (13).

## Anti-TPO-toxic-hypothyroidism

The auto-immune neuro-inflammation hypothesis is derived from the concept that persisting symptoms can be attributed to autoimmune disease rather than hypothyroidism *per se*. These patients report profound fatigue, poor sleep quality, muscle and joint tenderness, and dry mouth and eyes (14). In 2019 a surgical group conducted a definitive clinical trial (15) enlisting 150 patients with persistent Hashimoto-related symptoms and with extremely high anti-thyroperoxidase (anti-TPO) antibody titres (greater than 10 times the upper limit of normal). Five percent were on combination therapy or DTE. Total thyroidectomy achieved significantly improved quality of life scores and improved measures of fatigue as compared with conventional medical treatment. Sham surgery was not performed on the control group for ethical reasons. In any case such surgery would not mask the persistence of goitre in many of these subjects. The improvement was sustained at three to five years follow-up, militating against any substantial placebo response (16). The high complication rate was of concern: 4.1% had immediate post-operative infection. In the long-term 5.5% had recurrent laryngeal nerve palsy, and 4.1% had sustained hypocalcaemia.

A similar substantial improvement was reported in a purely observational study of total thyroidectomy performed on 154 Hashimoto disease patients who had persistent autoimmune symptoms (17). These patients were euthyroid with or without replacement therapy, and with *any* elevation of anti-TPO antibodies above the normal range. They were considered to be end-of-the-road patients in terms of available treatment options. The anti-TPO antibody titres were markedly reduced by surgery, but they did not predict the outcome. 1.9% had recurrent laryngeal nerve palsy and 3.9% residual hypoparathyroidism. A theoretical analysis suggests total thyroidectomy could be more cost effective than lifelong medical therapy (18). Most endocrinologists are likely to show extreme caution in recommending a technically difficult operation for a patient with diffuse non-specific symptoms.

The medical treatment for autoimmune symptoms in Hashimoto’s thyroiditis has received remarkably little attention and requires much more research. Anti-TPO antibody levels are reduced by selenium (19) and metformin (20) and also by corticosteroids (21). There is as yet no evidence of any clinical benefit from these agents, although it is noteworthy that Hashimoto’s disease complicated by encephalopathy is steroid-responsive (22) and can also improve with intravenous immunoglobulin (23).

## Hypothyroidism-in-denial

This diagnosis use made after excluding other possible causes. These patients experience significant distress. Endocrinologists do not receive training in recognising medically not yet explained symptoms (MNYES). An empathetic approach, reassurance, counselling, support, and even cognitive behaviour therapy is recommended (2).

## Thyroxine allergy/sensitivity

Drug hypersensitivity reactions and allergies to levothyroxine are extremely rare (24). These patients self-present with quite clear symptoms and are appropriately treated. The majority are adverse drug reactions causing palpitations, nausea, vomiting, and tremor. Allergies due to components such as fillers, excipients, or dyes may cause urticaria or angioedema. They can be confirmed by skin testing or a drug provocation test (25).

## Conclusion

These recommendations are made on the basis of clinical experience. There is an obvious need for confirmation by independent clinical reports. Clinical experience is the foundation upon which evidence-based medicine is built (26). It integrates individual clinical expertise with the best external evidence, and it paves the way for future research. The guidelines for treatment of hypothyroidism were based on the clinical experience of physicians who firmly recommended thyroxine monotherapy as the standard of care. The clinical experience of many European physicians has confirmed the psychosocial burden that exists in many but not all of these symptomatic hypothyroid patients.

More evidence is needed to achieve a consensus among physicians about this population of distressed hypothyroid patients and the subgroups that exist within it. Naming these subgroups should help future discussions. Some clinicians may think of better eponyms than the ones I have suggested. There must be vast untapped information in the case histories of endocrinologists and in the records of the larger thyroid clinics that could help to better define these categories described. Clinical trials with more careful selection criteria must be designed.

A final word: It is time to put the patellar hammer back in the pockets of Endocrinologists.

## Data Availability

The datasets presented in this article are not readily available because the article reflects my clinical experience and no data is provided at present. Requests to access the datasets should be directed to Prof Tim Welborn.
